# Latent Class Profiles of Police Violence Exposure in 4 US Cities and Their Associations with Anticipation of Police Violence and Mental Health Outcomes

**DOI:** 10.1007/s11524-022-00643-5

**Published:** 2022-06-06

**Authors:** Leslie Salas-Hernández, Jordan E. DeVylder, Hannah L. F. Cooper, Catherine dP Duarte, Alyasah A. Sewell, Elizabeth Reisinger Walker, Regine Haardörfer

**Affiliations:** 1 Rollins School of Public Health, Emory University, Atlanta, GA USA; 2 Graduate School of Social Work, Fordham University, New York, NY USA; 3 School of Medicine, Stanford University, Stanford, CA USA; 4 Department of Sociology, Emory University, Atlanta, GA USA

**Keywords:** Police violence, Latent class analysis, Mental health, Direct exposure to violence, Vicarious exposure to violence

## Abstract

While studying polyvictimization is well established within the broader violence literature and applied to other types of violence, it has yet to be documented whether polyvictimization also presents in patterns of police violence exposure (i.e., neglectful, psychological, physical, and sexual police violence). Our objective was to analyze latent patterns of co-occurring police contact and their associations with mental health. By applying latent class analysis (LCA) methods to the 2016 and 2017 Surveys of Police-Public Encounters (*N* = 2615), conducted in 4 Northeastern US cities, we identified classes of direct and vicarious police violence and compared sociodemographic characteristics among classes using multinomial regression. Classes were regressed on mental health outcomes. LCA identified four classes of police contact. Compared to Positive Police Contact (33.0%) class members, members of the (a) Extreme Police Violence (4.0%) class reported higher anticipation of future police victimization, psychological distress, and suicide ideations and attempts; they were more likely to be Black, cisgender men, and Latinx; (b) members of the High Police Violence (23.6%) class reported higher anticipation of future police victimization and psychological distress; they were more likely to be Black, Native American, and multiracial; members of the (c) Low Police Contact (39.5%) class had comparable mental health outcomes; they were more likely to report a household income < $19,999. Notably, no participants were unexposed to police contact. Polyvictimization presents in experiences of police violence and disproportionately impacts structurally marginalized people.

**Supplementary Information:**

The online version contains supplementary material available at 10.1007/s11524-022-00643-5.

## Introduction

The violence of policing is a documented determinant of adverse health outcomes [1]. However, traditional approaches to the study of police violence often do not apply intersectional methods which can inform our understanding of the patterning of police violence. An intersectional framework allows us to fully contextualize the ways individuals experience oppression and/or privilege during policing encounters [2, 3]. Applications of intersectional quantitative methods (e.g., latent class analysis) can identify patterns of co-occurring forms of police violence while incorporating an individual’s multiple intersecting identities (e.g., race, ethnicity, gender, sexuality, and nativity) [4]. Latent class analysis (LCA) identifies groups of individuals with common exposure patterns by using a person-centered modeling approach instead of a traditional variable-centered modeling approach (i.e., focusing on individual sociodemographic variables) [4]. The LCA can identify whether clusters of multiple forms of violence, or “polyvictimization,” have distinct relationships with health outcomes. While studying polyvictimization is well established within the broader violence literature and applied to other types of violence (e.g., intimate partner violence), it has yet to be documented whether polyvictimization also presents in patterns of police violence exposure [5, 6]. Forms of violence tend to co-occur across the life course but the police violence literature tends to focus on single types of police violence separately (e.g., physical police violence) instead of accounting for the clustering of police violence exposures (i.e., neglectful, psychological, physical, and sexual police violence) [7, 8].

Indirect, or vicarious, exposure to police violence also contributes to poor mental health. Trauma does not need to be personally experienced to affect mental health, though few studies have examined the prevalence and health consequences of vicarious exposure to police violence [9]. Additionally, expecting or anticipating racism results in psychological and cardiovascular responses independent of reports of direct experiences of racism [10, 11]. Research on the relationship between police violence and mental health has focused on depression and psychological distress outcomes, but the *anticipation* of future police victimization may also contribute to poor mental health and remains an understudied area [12].

Multiple forms of violence, for example, unfolded during George Floyd’s 20th, final, and fatal police encounter. In addition to the *direct physical* police violence experienced when Mr. Floyd was thrown and pinned to the ground for 9 min and 29 s, Mr. Floyd was also exposed to *direct psychological* police violence when officers taunted him to get in the police car but made no move to get off him. Others also experienced multiple forms of police violence during this single encounter. Ms. Darnella Frazier, the teenager who recorded it, was subjected to *vicarious physical* police violence. Bystanders who asked the police to check Mr. Floyd’s pulse experienced *direct psychological* police violence when an officer shook his mace spray at them. Though they were not present, this encounter affected Mr. Floyd’s daughter and loved ones lives in the forms of *vicarious physical* and *psychological* police violence. Moreover, past police encounters can reverberate through the present: before the police murdered Mr. Floyd, they had stopped him at least 19 times during adulthood [13], and these repeated encounters collectively form *direct psychological* police violence [7]. In previous studies, each of these forms of police violence have been studied separately and independently, therefore neglecting the potential impact of co-occurring violence.

To advance the field’s documentation of how different forms of police violence co-occur and their collective implications for health, we use latent class analysis (LCA), an intersectional, person-centered analytical approach [14, 15]. LCA identifies classes or patterns of exposure to policing, identifies who is most targeted by these forms of police violence, and assesses whether these policing exposure classes are associated with health outcomes. Our study also draws on the broader racism literature by incorporating anticipation of future police victimization as a vital outcome. Specifically, by leveraging the 2016 and 2017 Surveys of Police-Public Encounters, this study expands our understanding of exposure to police violence by the following:

1. Identifying differential patterns of direct and vicarious exposure to policing.
2. Describing characteristics of participants within each identified pattern of policing exposure.
3. Assessing the relationship between patterns of exposure to policing and anticipation of police violence.
4. Examining the relationship between patterns of exposure to policing and mental health outcomes.

## Methods

### Sample

We combined two datasets for this study: the 2016 Survey of Police-Public Encounters (SPPE I; *N* = 1615) and the 2017 Survey of Police-Public Encounters II (SPPE II; *N* = 1000), resulting in a total sample size of *N* = 2615 participants. The SPPE I was conducted in four US cities—Baltimore, NYC, Philadelphia, and Washington, DC during March–April 2016. The SPPE II was conducted in Baltimore and NYC during October–December 2017. Participants were recruited through Qualtrics Panels, an online survey administration service and sampled so that they were within + / − 10% of 2010 census distributions of age, sex, and race/ethnicity in each city. Further sampling details can be found in DeVylder et al. [16, 17].

### Measures

#### ***Exposure to Policing***

We used twelve dichotomous (yes/no) indicators from the Police Practices Inventory (PPI) of the SPPE I/II to assess self-reported *direct* and *vicarious* experiences of police contact. Police violence types assessed were neglectful, physical, physical with a weapon, psychological, and sexual. The police violence items were informed by prior qualitative work [7]. Ten of those items captured direct (e.g., “has a police officer ever hit, punched…used physical force against *you*?”) and vicarious (e.g., “has a police officer ever hit, punched…used physical force against a *close friend* or *family member*) police violence (Table 1). Two of those items assessed direct and vicarious positive police contact.

**Table 1:** Types of police contact

	Type of police contact	Item (yes/no)
Direct exposure	Neglectful police violence	Have you ever called or summoned the police for assistance and the police either did not respond, responded too late, or responded inappropriately?
	Physical police violence	Has a police officer ever hit, punched, kicked, dragged, beat, or otherwise used physical force against you?
	Physical police violence with a weapon	Has a police officer ever used a gun, baton, taser, or other weapon against you?
	Psychological police violence	Has a police officer ever engaged in non-physical aggression towards you, including threatening, intimidating, stopping you without probable cause, or using slurs?
	Sexual police violence	Has a police officer ever forced inappropriate sexual contact on you, including while conducting a body search in a public place?
	Positive police contact	Has a police officer ever provided assistance, protection, or any other service to you?
Vicarious (or indirect) exposure	Neglectful police violence	Has a close friend or family member ever called or summoned the police for assistance and the police either did not respond, responded too late, or responded inappropriately?
	Physical police violence	Has a police officer ever hit, punched, kicked, dragged, beat, or otherwise used physical force against a close friend or family member?
	Physical police violence with a weapon	Has a police officer ever used a gun, baton, taser, or other weapon against a close friend or family member?
	Psychological police violence	Has a police officer ever engaged in non-physical aggression towards a close friend or family member, including threatening, intimidating, stopping him or her without probable cause, or using slurs?
	Sexual police violence	Has a police officer ever forced inappropriate sexual contact on a close friend or family member, including while conducting a body search in a public place?
	Positive police contact	Has a police officer ever provided assistance, protection, or any other service to a close friend or family member?

#### Outcomes

This analysis had two primary sets of outcomes. The first outcome, anticipation of police violence, was measured using the 8-item Expectation of Police Practices Scale (EPPS). The EPPS assesses self-reported *expectations* of direct and vicarious police violence (e.g., “against you or a close friend or family member”) in the next year. Participants responded using a 4-point Likert scale: not at all likely (0), unlikely (1), likely (2), and almost certainly (3). Summed scores ranged from 0 to 24. The Cronbach’s *α* for SPPE I and SPPE II were both excellent (*α* = 0.95 for both).

We also examined a set of mental health outcomes, including psychological distress, suicidal ideation, and suicide attempt. Psychological distress during the last 30 days was assessed using the 6-item Kessler Psychological Distress Scale (K-6). Participants responded using a 5-point scale: none of the time (0), a little of the time (1), some of the time (2), most of the time (3), and all of the time (4). Summed scores ranged from 0 to 24. The Cronbach’s *α* for psychological distress for both SPPE I and SPPE II were excellent (*α* = 0.90 and *α* = 0.92, respectively). SPPE I/II assessed past-year suicidal ideation and attempt and offered three answer choices for each: yes, no, and unsure. For analysis, we dichotomized responses by combining the “unsure” and “no” options for each indicator [17].

#### Covariates

We used the following variables in the analysis: gender/sex, race, ethnicity, sexual orientation, nativity status, income, education, and age. Age brackets were aligned with Bui et al.’s (2018) article on police killings and years of life lost (YLLs) [18]. Having previously engaged in criminalized activities was not assessed consistently across datasets, so the items were combined using the following categories—drug use, theft, and causing injury to someone (Table 2). Each of the three items was dichotomized into “no self-reported prior engagement in the criminalized activity” and “reported prior engagement in the criminalized activity.” When testing for associations between the exposure to policing and outcomes, we also adjusted for these covariates as potential confounders.

**Table 2:** Observed distribution of sociodemographic and sociobehavioral characteristics in the overall sample

	*N* = 2615 (100%)
Direct police encounters	*n* (%)
Neglectful police violence	525 (20.1)
Physical police violence	216 (8.3)
Physical Police Violence with a Weapon	119 (4.6)
Psychological police violence	516 (19.8)
Sexual police violence	88 (3.4)
Positive policing	1293 (49.4)
Vicarious police encounters	
Neglectful police violence	427 (16.3)
Physical police violence	493 (18.9)
Physical Police Violence with a Weapon	304 (11.6)
Psychological police violence	624 (24.0)
Sexual police violence	103 (3.9)
Positive policing	1271 (48.6)
Mental health	
Mental health diagnosis	568 (22.0)
Anticipation of future police victimization	6.20 (5.97)
Psychological distress	5.30 (5.62)
Suicide ideations	240 (9.4)
Suicide attempts	54 (2.1)
	Mean (SD)
Age (continuous)	39.6 (14.9)
	N (col%)
Age (categorical)^a^	
18–24	437 (16.7)
25–34	734 (28.1)
35–44	503 (19.2)
45–54	439 (16.8)
55 +	502 (19.2)
Gender/sex^b^	
Cisgender men	1066 (40.8)
Cisgender women	1532 (58.6)
Transgender and other genders	17 (0.7)
Race	
Asian/Pacific Islander	109 (4.2)
Black/African American	917 (35.1)
Native American	43 (1.6)
Multiracial or other race	139 (5.3)
White	1407 (53.8)
Latino ethnicity	
Latinx	362 (13.9)
Non-Latinx	2242 (86.1)
Sexual orientation	
Bisexual	136 (5.2)
Gay/lesbian/homosexual	94 (3.6)
Heterosexual/straight	2334 (89.5)
Sexual orientation not specified	45 (1.7)
Nativity	
Born in USA	2346 (90.1)
Not born in USA	258 (9.9)
Household income	
< $19,999	372 (14.3)
$20,000–39,999	483 (18.5)
$40,000–59,999	499 (19.1)
$60,000–79,999	436 (16.7)
$80,000–99,999	283 (10.9)
$100,000 +	533 (20.5)
Education	
Did not complete high school	74 (2.8)
High school diploma or GED	496 (19.0)
Some college or technical school	739 (28.3)
College graduate	877 (33.6)
Graduate or professional degree	423 (16.2)
Criminalized activities	
Buying drugs, selling drugs, using heroin, using injected opiate drugs^c^ or selling marijuana^d^	366 (14.0)
Stealing^e^, robbing, burglarizing someone’s property^c^ or taking money or goods	205 (7.8)
Assaulting someone^c^ or injuring someone in a fight^d^	340 (13.0)

^a^Age brackets were aligned with Bui et al.’s 2018 article on police killings and years of life lost (YLLs) [18]. ^b^While the survey question asked participants to indicate their self-identified gender, the answer options corresponded to biological sex. Moving forward, the authors will use the cisgender man and cisgender woman to describe the participants who selected male and female, respectively. ^c^The 2016 Survey of Police-Public Encounters (SPPE I); ^d^The 2017 Survey of Police-Public Encounters (SPPE II); ^e^SPPE I and SPPE II

### Data Analysis

Univariate analyses were conducted to characterize the sample. Bivariate analysis assessed relationships between the police contact classes resulting from the LCA and the identified covariates. Specific analyses are presented by the study’s research questions. All analyses were conducted with SPSS Version 26 [19] and Mplus 8 [20].

#### What Are the Distinct Patterns of Direct and Vicarious Policing?

The LCA was conducted using a total of 12 dichotomous police encounter indicators, or “manifest” variables, from the PPI [21]. Since forced-choice responding was utilized for most of the survey items, missing data was not a concern. A succession of models using the selected indicators was analyzed starting with a 1-class model. Model fit was assessed by relative fit indicators between models including Akaike Information Criterion (AIC), Bayesian Information Criterion (BIC), and the sample-size adjusted BIC (SABIC), entropy values, and by the Vuong-Lo-Mendell-Rubin Likelihood Ratio Test. To confirm the model solution for types of police contact, we re-ran the LCA solely using SPPE I, the larger dataset.

#### How Do Sociodemographic Characteristics and Sociobehavioral Experiences Vary across Police Contact Classes?

After defining the police contact classes, we used multinomial regressions to compare sociodemographic characteristics and sociobehavioral experiences across classes. Among the resulting police contact classes (described below in the Results section), we use the “Positive Police Contact” class as the referent, given that there are no participants in the study who were completely unexposed to police contact (the ideal referent). The reference group for each sociodemographic characteristic or sociobehavioral experience was the category believed to have the least exposure to police violence.

#### How Are Police Contact Classes Associated with Anticipation of Police Violence and Mental Health?

The associations between class membership and “anticipation of police violence” were explored using linear regression. We explored the associations between policing patterns and mental health outcomes using linear regression (for the continuous outcome: psychological distress) and logistic regression (for the dichotomized outcomes: suicidal ideation and suicide attempt). We present unadjusted results, as well as results adjusted for race, Latinx ethnicity, gender, age, education, income, sexual orientation, nativity, and prior engagement with criminalized activities.

## Ethics

Emory University’s Institutional Review Board approved this secondary data analysis.

## Results

### Sample Characteristics

Participants (*N* = 2615) were aged 18 to 95 years old (mean = 39.6 years; SD = 14.9) (Table 2). Lifetime exposure to direct police contact varied: 20.1% of participants reported experiences of neglectful police violence; 8.3% reported experiences of physical police violence; 4.6% reported experiences of physical police violence with a weapon; 19.8% reported experiences of psychological police violence; 3.4% reported experiences of sexual police violence; and 49.4% reported experiences of positive police contact. Mean anticipation of future police victimization was 6.20 (SD = 5.97;) and mean self-reported psychological distress was 5.30 (SD = 5.62;). A smaller proportion reported suicidal ideation (9.4%) or suicide attempt (2.1%) in the year prior to completing the survey.

### What Are the Distinct Police Contact Classes of Direct and Vicarious Policing?

After comparing models with 1 to 7 latent classes, we identified the 4-class model based on the highest entropy (Supplementary Table 1). While the AIC, BIC, and SAIBIC were gradually decreasing as the number of classes in the model increased, 5 classes did not produce a meaningful difference from 4 classes as indicated by the Vuong-Lo-Mendell-Rubin Likelihood Ratio Test. To confirm these findings, we ran the LCA solely using SPPE I, the larger dataset; four distinct classes were found with similar patterns.

**Fig. 1:**
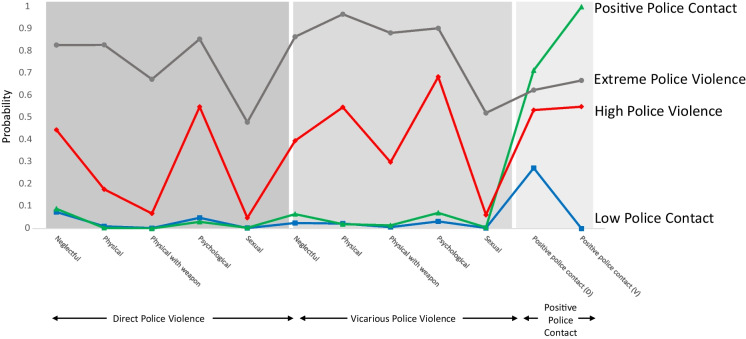
Latent classes and probability of each form of police encounter. Note: The connecting lines show the patterns of exposure probabilities; they do not represent a relationship/trajectory connecting the dots. D, direct; V, vicarious. Naming these classes of exposure warranted discussion and we decided not to use the terms “moderate” or “medium” to describe what we currently refer to as the “High Police Violence” class because we did not want to indivertibly contribute to the normalization of this form of violence

We labeled the four classes as: Extreme Police Violence, High Police Violence, Low Police Contact, and Positive Police Contact. A total of 105 participants (4.0%) reported experiences of “Extreme Police Violence;” 616 participants (23.6%) reported experiences of “High Police Violence;” 1032 participants (39.5%) reported experiences of “Low Police Contact;” and 862 participants (33.0%) reported experiences of “Positive Police Contact.” LCA is a person-centered analysis approach that estimates classes of exposure in the general population and the probability of each exposure due to class membership. Thus, we expect that 4% of the study population have been exposed to “Extreme Police Violence” with probabilities of experiencing the following: direct neglectful (82.8%) and vicarious neglectful (86.5%), direct physical (82.9%) and vicarious physical (96.7%); direct psychological (85.5%) and vicarious psychological (90.3%), and direct sexual (48.0%) and vicarious sexual (52.1%) police violence; as well as direct positive police contact (62.5%) and vicarious positive police contact (66.8%). Figure 1 displays the relationship between latent class membership and the estimated probabilities of the 12 police contact indicators, or “manifest” variables.

### How Do Sociodemographic Characteristics and Sociobehavioral Experiences Vary across Police Contact Classes?

Our multinomial regression analysis identified sociodemographic and sociobehavioral differences across the police contact classes. While we describe statistically significant findings here, we present all results in Table 3. Compared to participants in the “Positive Police Contact” class, participants in the “Extreme Police Violence” class were more likely to identify as cisgender men (*OR*: 2.48, 95% *CI*: 1.54–3.97; *p* < 0.001), Black (*OR*: 3.83, 95% *CI*: 2.25–6.52; *p* < 0.001), and Latinx (*OR*: 2.24, 95% *CI*: 1.21–4.13; *p* < 0.05). Compared to the participants in the “Positive Police Contact” class, participants in the “High Police Violence” class were more likely to identify as Black (*OR*: 3.15, 95% *CI*: 2.43–4.08; *p* < 0.001), Native American (*OR*: 2.89, 95% *CI* = 1.32–6.32; *p* < 0.01), and multiracial (*OR*: 2.27, 95% *CI*: 1.36–3.78; *p* < 0.01). In comparison to participants in the “Positive Police Contact” class, participants in the “Low Police Contact” class were more likely to report a household income < $19,999 (*OR*: 1.77, 95% *CI*: 1.23–2.56; *p* < 0.01). Compared to participants in the “Positive Police Contact” class, both participants in the “High Police Violence” and “Extreme Police Violence” classes were more likely to report prior engagement with criminalized activities such as drug use and having injured someone (getting in a fight or assaulting someone). Additionally, when compared to participants in the “Positive Police Contact” class, participants in the “Extreme Police Violence” or “High Police Violence” classes were less likely to have been born in the US (Supplementary Table 3).

**Table 3:** Sociodemographic and sociobehavioral characteristics of class membership compared to the “Positive Policing” Class using multinomial logistic regression (*N* = 2549)

	Low Police Contact *N* = 1014				High Police Violence *N* = 598				Extreme Police Violence *N* = 101			
Covariate	*OR*	95% *CI*		*p*-value	*OR*	95% *CI*		*p*-value	*OR*	95% *CI*		*p*-value
Age												
18–24	0.68	0.50	0.94	< 0.05	1.80	1.18	2.75	< 0.05	5.45	1.18	25.08	< 0.01
25–34	0.78	0.59	1.02	0.07	2.48	1.69	3.62	< 0.01	11.54	2.67	49.96	< 0.01
35–44	0.75	0.56	1.01	0.06	1.96	1.31	2.92	< 0.01	10.76	2.45	47.30	< 0.01
45–54	0.77	0.57	1.03	0.08	1.53	1.01	2.32	< 0.05	5.31	1.13	24.85	< 0.05
55 + (ref)	-	-	-	-	-	-	-	-	-	-	-	-
Gender												
Cisgender man	0.91	0.75	1.10	0.34	1.05	0.83	1.32	0.68	2.48	1.54	3.97	< 0.001
Cisgender woman (ref)	-	-	-	-	-	-	-	-	-	-	-	-
Race												
Asian/Pacific Islander	1.48	0.92	2.38	0.10	1.11	0.57	2.17	0.75	1.24	0.26	5.93	0.79
Black/African American	1.18	0.94	1.48	0.15	3.15	2.43	4.08	< 0.001	3.83	2.25	6.52	< 0.001
Native American	0.67	0.29	1.56	0.35	2.89	1.32	6.32	< 0.01	1.87	0.35	9.89	0.46
Multiracial or other race	0.97	0.61	1.56	0.91	2.27	1.36	3.78	< 0.01	1.91	0.70	5.20	0.21
White (ref)	-	-	-	-	-	-	-	-	-	-	-	-
Latino ethnicity												
Latinx	1.03	0.76	1.40	0.83	1.16	0.82	1.64	0.40	2.24	1.21	4.13	< 0.05
Non-Latinx (ref)	-	-	-	-	-	-	-	-	-	-	-	-
Sexual orientation												
Bisexual	0.72	0.44	1.17	0.19	1.44	0.90	2.29	0.13	1.29	0.52	3.22	0.58
Gay/lesbian/homosexual	0.88	0.52	1.51	0.65	1.21	0.67	2.19	0.53	0.23	0.03	1.78	0.16
Sexual orientation not specified	1.10	0.53	2.26	0.80	0.83	0.33	2.09	0.69	0.85	0.10	7.13	0.88
Heterosexual/straight (ref)	-	-	-	-	-	-	-	-	-	-	-	-
Nativity												
Not born in USA	1.19	0.87	1.63	0.28	0.56	0.37	0.85	< 0.01	0.24	0.08	0.72	< 0.05
Born in USA (ref)	-	-	-	-	-	-	-	-	-	-	-	-
Household income												
< $19,999	1.77	1.23	2.56	< 0.01	1.49	0.94	2.37	0.09	1.59	0.66	3.86	0.30
$20,000–39,999	1.24	0.90	1.71	0.18	1.57	1.05	2.34	< 0.05	0.82	0.34	1.96	0.65
$40,000–59,999	1.19	0.88	1.61	0.27	1.43	0.97	2.10	0.07	1.08	0.49	2.40	0.85
$60,000–79,999	1.24	0.92	1.68	0.17	1.59	1.08	2.34	< 0.05	0.91	0.38	2.13	0.82
$80,000–99,999	0.90	0.64	1.27	0.56	1.32	0.86	2.02	0.21	1.83	0.79	4.21	0.16
$100,000 + (ref)	-	-	-	-	-	-	-	-	-	-	-	-
Education												
Did not complete high school	2.29	1.06	4.96	< 0.05	1.76	0.74	4.20	0.20	3.76	0.91	15.46	0.07
High school diploma or GED	0.88	0.62	1.25	0.47	0.75	0.49	1.15	0.18	1.43	0.55	3.71	0.46
Some college or technical school	0.92	0.67	1.24	0.57	0.94	0.64	1.37	0.73	1.68	0.68	4.11	0.23
College graduate	1.01	0.76	1.33	0.95	1.00	0.70	1.42	1.00	1.55	0.67	3.57	0.30
Graduate or professional degree (ref)	-	-	-	-	-	-	-	-	-	-	-	-
Criminalized activities												
Bought or sold drugs; used heroin or injected opiate drugs (ref: did not engage in activity)	0.78	0.54	1.12	0.18	1.89	1.32	2.70	< 0.001	3.11	1.72	5.62	< 0.001
Stole, robbed, or burglarized someone; taken money or goods (ref: did not engage in activity)	0.83	0.49	1.40	0.47	1.12	0.68	1.83	0.67	1.36	0.67	2.73	0.39
Assaulted someone; injured someone in a fight (ref: did not engage in activity)	0.97	0.65	1.44	0.87	2.10	1.43	3.08	< 0.001	4.83	2.67	8.74	< 0.001

Transgender and participants with other genders were excluded from regression analysis due to the small sample size. These multinomial regressions estimated comparisons to the privileged group for each covariate of interest. For example, for sexual orientation, individuals who identify as straight/heterosexual belong to a privileged group and were thus the reference category

### How Are Police Contact Classes Associated with Anticipation of Police Violence?

When compared to the “Positive Police Contact” class, participants in the “Extreme Police Violence” class had, on average, 10.32 more points (SE = 0.52) while participants in the “High Police Violence” class had, on average, 5.60 more points (SE = 0.26) on the expectation of future police victimization scale (Table 4). Participants in the “Low Police Contact” class did not meaningfully differ from participants in the “Positive Police Contact” class on anticipated future police violence (b = 0.16, SE = 0.22).

**Table 4:** Unadjusted and fully adjusted regression models of the association between LCA class and expectations of future police violence (EPPS total score)

	Expectation of future police violence (EPPS total score) b (SE)	
	Unadjusted *N* = 2548	Adjusted *N* = 2487
Positive policing (ref)	-	-
Low police contact	0.34 (0.23)	0.16 (0.22)
High police violence	6.78 (0.26)***	5.60 (0.26)***
Extreme police violence	12.32 (0.51)***	10.32 (0.52)***

Adjusted for race, Latinx ethnicity, gender, age, education, income, sexual orientation, nativity, and involvement in criminalized activities; *p < 0.05; **p < .01; ***p < .001

### How Are Police Contact Classes Associated with Mental Health?

Members of the “Extreme Police Violence” class scored, on average, 2.63 points (SE = 0.55) higher on the psychological distress scale compared to members of the “Positive Police Contact” class (Table 5). The “Extreme Police Violence” class was associated with 3.01 times the odds (95% *CI*: 1.64–5.53) of reporting suicidal ideation and 8.54 times the odds (95% *CI*: 2.79–26.12) of reporting suicide attempt compared to the “Positive Police Contact.” Similarly, compared to those in the ‘Positive Police Contact,” “High Police Violence” was associated with a higher odds of reporting suicidal ideation (*OR*: 1.48, 95% *CI*: 0.99–2.20) and suicide attempt (*OR*: 2.20, 95% *CI*: 0.81–6.00) though once adjusted the results included the null. Finally, those in the “Low Police Contact” class scored, on average, 0.55 points (SE = 0.25) lower on the psychological distress scale compared to members of the “Positive Police Contact” class though once adjusted the results included the null.

**Table 5:** Unadjusted and fully adjusted regression models of the association between LCA class and mental health outcomes

	Psychological distress (K-6 total score) b (SE)		Suicidal ideation *OR* (95% *CI*)^1^		Suicide attempts *OR* (95% *CI*)	
	Unadjusted *N* = 2588	Adjusted *N* = 2523	Unadjusted *N* = 2614	Adjusted *N* = 2548	Unadjusted *N* = 2614	Adjusted *N* = 2548
Positive policing (ref)	-	-	1.0	1.0	1.0	1.0
Low police contact	− 0.55 (0.25)*	− 0.42 (0.23)	0.92 (0.64–1.33)	0.96 (0.65–1.43)	1.25 (0.44–3.54)	1.40 (0.48–4.03)
High police violence	2.32 (0.29) ***	1.16 (0.28) ***	2.31 (1.63–3.28)***	1.48 (0.99–2.20)	4.78 (1.91–11.98)**	2.20 (0.81–6.00)
Extreme police violence	5.01 (0.57) ***	2.63 (0.55) ***	5.55 (3.36–9.14)***	3.01 (1.64–5.53)***	31.48 (12.25–80.94)***	8.54 (2.79–26.12)***

Adjusted for race, Latinx ethnicity, gender, age, education, income, sexual orientation, nativity, and involvement in criminalized activities; *p < 0.05; **p < .01; ***p < .001

^1^Example interpretation: Those in the “Extreme Police Violence” class were associated with 3.01 times the odds (95% *CI*: 1.64–5.53) of reporting suicidal ideation compared to those in the “Positive Police Contact” class

## Discussion

This study was the first to explore and show variation in direct and vicarious police encounters through a person-centered analysis, explore sociodemographic characteristics and sociobehavioral experiences of these classes, and investigate their implications for the anticipation of future police violence and mental health.

### Distinct Classes of Direct and Vicarious Exposure to Police Contact

Exposure to direct and vicarious police violence is integrated into certain people’s lives, while it is nonexistent for others. Approximately 70% of the participants were in the “Positive Police Contact” or “Low Police Contact” classes. People in these classes had been insulated from police violence: neither they nor their close friends or family members experienced police violence. A striking 30% of participants, however, were in the “Extreme Police Violence” and “High Police Violence” classes. Of note, our findings indicate that both the “Extreme Police Violence” and “High Police Violence” groups have been exposed to high levels of policing overall, including experiences of positive policing—both directly and vicariously. This is a notable observation as it suggests that positive policing interventions are insufficient to address police violence. Specifically, this finding aligns with legal theory, which emphasizes that higher levels of overall police contact heighten risk for police violence and deaths caused by police [22]. Together with these legal findings, our research suggests that shifting to community policing strategies—which increase overall police contact—may not reduce police violence. Additionally, the “Extreme” and “High Police Violence” classes reported high probabilities of nearly each form of vicarious police violence. While vicarious exposure to police killings have been shown to negatively impact Black mental health [23, 24], the effects of vicarious *non-fatal* police violence may also “spillover” to mental health outcomes. While this study examined direct and vicarious police violence exposure, future research can utilize LCA to assess classes of exposure across overlapping forms of violence (e.g., intimate partner violence and police violence).

### Different Policing for Different People

Our person-centered LCA analysis revealed racial and ethnic differences across policing exposure classes. Black participants were more likely to be in the “Extreme Police Violence” and “High Police Violence” classes when compared to White people. While several other studies have shown that Black people are disproportionately affected by police use of force, this shows that Black people are more likely to have experienced multiple, co-occurring forms of both direct and vicarious police violence [14, 18, 25]. By contrast, White people made up the majority of the “Positive Police Contact” and “Low Police Contact” classes, with very few in the High and Extreme Police Violence classes. We also found that Native Americans are 2.89 times more likely to be in the “High Violence” group compared to White individuals. Data erasure often masks the experiences of Native Americans, so prior reports of police violence among Native Americans are likely undercounts [1]. Our findings also demonstrated that people born outside the USA are much less likely to be in the “High Police Violence” and “Extreme Police Violence” classes. Studies have found that compared to people born in the USA, immigrants are less likely to interact with the police regardless of their documentation status [26].

Cisgender men were 2.5 times more likely to be in the “Extreme Police Violence” class in comparison to cisgender women, but there were no gendered differences in the “High Police Violence” class. This diverges from the broader police violence literature, which often reports gendered differences in experiences of police violence with men at greater risk [14, 27]. However, that literature has not considered the co-occurrence of multiple forms of violence which may explain differences. While our study suggests women are equally likely to be exposed to “High Police Violence,” their stories are often not centered. Specifically, the “#SayHerName” movement has called attention to the erasure of Black women’s experiences of police violence in the national discourse. Of noteworthy are the twelve transgender participants who are included in our LCA analysis, and not in the subsequent analysis (i.e., regressions). We found that six of the transgender participants were in the “High Police Violence” class and one participant was in the “Extreme Police Violence” class. Historically, transgender and gender-nonconforming individuals report disproportionate contact with police and police violence [28]. Future police violence surveys should seek to utilize sample designs powered enough to analyze specifically or inclusively of transgender and nonbinary (i.e., individuals who identify outside the gender binary) populations, such as those recommended by Restar and colleagues, to ensure documentation of how they may be disproportionately impacted [29]. Participants in the “Extreme” and “High Police Violence” classes were more likely to report previous engagement in some criminalized activities—drug use and causing injury to someone. This aligns with the existing literature, which finds that people who engage in criminalized activities report more contact with police [1, 30]. Together with literature documenting that criminalized activities occur in equal rates across social groups, these findings are consistent with the notion that criminalization—of activities and identities—facilitates increased police contact [31, 32].

### Exposure to Police Violence Is Associated with Anticipation of Future Police Victimization

Qualitative studies have thoroughly described experiences of the anticipation or expectation of police violence and the resulting hypervigilance and avoidance of police; however, quantitative studies on this topic remain limited [10, 11, 33, 34]. Our study found that compared to participants in the “Positive Police Contact” class, participants in both the “Extreme Police Violence” and “High Police Violence” classes scored much higher on the expectations of future police victimization scale. We are not pathologizing anticipation of police violence, as it is a normal response for individuals who experience police violence, either directly or vicariously. Past research on expectations of discrimination indicates that anticipatory contact may hold important implications for mental and physical health outcomes that need to be explored in future research on police violence.

### Exposure to Police Violence Is Associated with Poor Mental Health

Our findings demonstrate significant and distinct associations between police violence exposure classes and mental health outcomes. These results build on DeVylder et al.’s findings of significant associations between each form of police violence and poor mental health outcomes [16]. By not assessing the co-occurrence of police violence exposure, the effects on mental health may have been underestimated [35]. Thus, we add findings that individuals in the “Extreme Police Violence” class had more severe mental health outcomes—higher psychological distress scores and more likely to report suicidal ideations and suicide attempts—while those in the “High Police Violence” classes had higher psychological distress scores.

### Limitations

Though this study found that policing exposure classes were associated with mental health outcome, it was cross-sectional and could not assess causality. Additionally, because the police violence measure assessed lifetime exposure to police contact, we are unable to assess trajectories of police violence exposure throughout the life course or whether these trajectories have different relationships to mental health. In this analysis, we assumed exposure to police violence predated the mental health outcomes; however, given the criminalization of mental health experiences, it is possible that people experiencing mental health crises may have more police encounters. Given all participants in this analysis had some contact with policing, we were unable to examine the health experiences of people with no contact to police. Additionally, we cannot be sure that a participant did not participate both in SPPE I and SPPE II. However, the average time of involvement in Qualtrics Panels (QP) is 6 months, so minimal overlap likely occurred since the time span between both surveys was 17 months. Lastly, these data were collected 3 and 4 years before the Black Lives Matter (BLM) Summer 2020 Protests, and we are unsure the degree to which these findings remain generalizable post-BLM protests.

## Public Health Implications

Consistent with the broader violence literature, our study found that polyvictimization presents in experiences of police violence, disproportionately impacting structurally marginalized people with implications for health inequity. These findings support a need to recognize and further investigate the forms of direct and vicarious police violence since classes of policing exposure may have distinct health implications. Future police violence research can incorporate person-centered analysis, which is already widely used in violence research. Enhanced public mental health efforts are needed since individuals who report high and extreme levels of police violence exposure may require nuanced mental health support. Our findings also strongly support calls by the American Public Health Association for structural interventions on exposure to policing and its health consequences [23]. As our nation grapples with redefining community safety, we need to acknowledge that the harms of police violence are compounded and widespread.

## Supplementary Information

Below is the link to the electronic supplementary material.

Supplementary file1 (DOCX 47 kb)
