# Supplementary material for: Latent Class Profiles of Police Violence Exposure in 4 US Cities and Their Associations with Anticipation of Police Violence and Mental Health Outcomes
Source: J Urban Health. 2022 Jun 6;99(4):655–68. doi: 10.1007/s11524-022-00643-5 (PMC9360379; doi:10.1007/s11524-022-00643-5)
Supplement: Supplementary file 1 — Supplementary file1 (DOCX 47 kb) [file 11524_2022_643_MOESM1_ESM.docx]

**Supplementary Table 1.** Model Fit Statistics for LCA (N = 2615)

| Classes | AIC | BIC | SABIC | Entropy | Vuong-Lo-Mendell-Rubin Likelihood Ratio Test |
| --- | --- | --- | --- | --- | --- |
| 1 | 26181.50 | 26251.92 | 26213.80 | - | - |
| 2 | 22240.94 | 22387.67 | 22308.24 | 0.88 | <0.01 |
| 3 | 21749.45 | 21972.48 | 21851.74 | 0.87 | <0.01 |
| 4 | 21385.59 | 21684.91 | 21522.87 | 0.90 | <0.01 |
| 5 | 21253.99 | 21629.61 | 21426.26 | 0.87 | 0.11 |
| 6 | 21146.13 | 21598.04 | 21353.39 | 0.87 | 0.31 |
| 7 | 21069.91 | 21598.13 | 21312.17 | 0.87 | 0.16 |

AIC = Akaike Information Criterion; BIC = Bayesian Information Criterion; SAIBIC = Sample-size adjusted Bayesian Information Criteria

**Supplementary Table 2.** Observed Distribution of Sociodemographic and Sociobehavioral Characteristics Within Each Latent Class

|  | Class | | | | | | | |
| --- | --- | --- | --- | --- | --- | --- | --- | --- |
|  | Low Police Contact | | Positive Policing | | High Police Violence | | Extreme Police Violence | |
|  | N=1032  (39.5%) | | N=862  (33.0%) | | N=616  (23.6%) | | N=105  (4.0%) | |
|  | Mean | SD | Mean | SD | Mean | SD | Mean | SD |
| **Age (continuous)** | 41.8 | 15.7 | 40.6 | 15.3 | 35.3 | 12.5 | 33.7 | 9.2 |
|  | n | col% | n | col% | n | col% | n | col% |
| **Age (categorical)** |  |  |  |  |  |  |  |  |
| 18-24 | 156 | 15.1 | 142 | 16.5 | 119 | 19.3 | 20 | 19.0 |
| 25-34 | 253 | 24.5 | 219 | 25.4 | 221 | 35.9 | 41 | 39.0 |
| 35-44 | 183 | 17.7 | 162 | 18.8 | 129 | 20.9 | 29 | 27.6 |
| 45-54 | 179 | 17.3 | 159 | 18.4 | 89 | 14.4 | 12 | 11.4 |
| 55+ | 261 | 25.3 | 180 | 20.9 | 58 | 9.4 | 3 | 2.9 |
| **Gender/Sex** |  |  |  |  |  |  |  |  |
| Cisgender Men | 397 | 38.5 | 351 | 40.7 | 251 | 40.7 | 67 | 63.8 |
| Cisgender Women | 633 | 61.3 | 506 | 58.7 | 358 | 58.1 | 35 | 33.3 |
| Transgender and other genders | 2 | 0.2 | 5 | 0.6 | 7 | 1.1 | 3 | 2.9 |
| **Race** |  |  |  |  |  |  |  |  |
| Asian/Pacific Islander | 55 | 5.3 | 36 | 4.2 | 15 | 2.4 | 3 | 1.9 |
| Black/African American | 321 | 31.1 | 229 | 26.6 | 312 | 50.6 | 55 | 52.4 |
| Native American | 10 | 1.0 | 14 | 1.6 | 17 | 2.8 | 2 | 2.9 |
| Multiracial or other race | 47 | 4.6 | 41 | 4.8 | 44 | 7.1 | 7 | 6.7 |
| White | 599 | 58.0 | 542 | 62.9 | 228 | 37.0 | 38 | 36.2 |
| **Latino Ethnicity** |  |  |  |  |  |  |  |  |
| Latinx | 127 | 12.3 | 111 | 13.0 | 98 | 16.0 | 26 | 24.8 |
| Non-Latinx | 904 | 87.7 | 746 | 87.0 | 513 | 84.0 | 79 | 75.2 |
| **Sexual Orientation** |  |  |  |  |  |  |  |  |
| Bisexual | 34 | 3.3 | 41 | 4.8 | 53 | 7.6 | 8 | 8.6 |
| Gay/Lesbian/Homosexual | 33 | 3.2 | 30 | 3.5 | 29 | 1.9 | 2 | 4.7 |
| Heterosexual/Straight | 943 | 91.5 | 773 | 90.1 | 525 | 88.6 | 93 | 85.4 |
| Sexual orientation not specified | 21 | 2.0 | 14 | 1.6 | 8 | 1.9 | 2 | 1.3 |
| **Nativity** |  |  |  |  |  |  |  |  |
| Born in USA | 902 | 87.7 | 774 | 90.2 | 570 | 93.0 | 100 | 95.2 |
| Not born in USA | 126 | 12.3 | 84 | 9.8 | 43 | 7.0 | 5 | 4.8 |
| **Household Income** |  |  |  |  |  |  |  |  |
| < $19,999 | 166 | 16.1 | 89 | 10.4 | 94 | 15.3 | 23 | 21.9 |
| $20,000 – 39,999 | 183 | 17.8 | 148 | 17.3 | 137 | 22.3 | 15 | 14.3 |
| $40,000 – 59,999 | 192 | 18.6 | 160 | 18.7 | 126 | 20.5 | 21 | 20.0 |
| $60,000 – 79,999 | 174 | 16.9 | 138 | 16.1 | 109 | 17.8 | 15 | 14.3 |
| $80,000 – 99,999 | 97 | 9.4 | 108 | 12.6 | 64 | 10.4 | 14 | 13.3 |
| $100,000 + | 218 | 21.2 | 214 | 25.0 | 84 | 13.7 | 17 | 16.2 |
| **Education** |  |  |  |  |  |  |  |  |
| Did not complete high school | 34 | 3.3 | 13 | 1.5 | 21 | 3.4 | 6 | 5.7 |
| High school diploma or GED | 187 | 18.2 | 156 | 18.1 | 125 | 20.3 | 28 | 26.7 |
| Some college or technical school | 285 | 27.7 | 240 | 27.9 | 184 | 19.9 | 30 | 28.6 |
| College graduate | 347 | 33.8 | 295 | 34.3 | 203 | 33.0 | 32 | 30.5 |
| Graduate or professional degree | 175 | 17.0 | 157 | 18.2 | 82 | 13.3 | 9 | 8.6 |
| **Criminalized Behaviors** |  |  |  |  |  |  |  |  |
| Buying drugs, selling drugs, using heroin, using injected opiate drugs^a^ or selling marijuana^b^ | 80 | 7.8 | 89 | 10.4 | 147 | 23.9 | 49 | 46.7 |
| Stealing^c^, robbing, burglarizing someone’s property^a^ or taking money or goods | 39 | 3.8 | 44 | 5.1 | 87 | 14.1 | 35 | 33.3 |
| Assaulting someone^a^ or injuring someone in a fight^b^ | 73 | 7.1 | 67 | 7.8 | 146 | 23.7 | 54 | 51.9 |

Notes: ^a^ SPPE I; ^b^ SPPE II; ^c^ SPPE I and SPPE II

**Supplementary Table 3.** Bivariate analysis of sociodemographic and sociobehavioral characteristics of class membership compared to the “Positive Policing” Class using multinomial logistic regression (N=2615)

|  | Low Police Contact  **N=1014** | | | | High Police Violence  **N=598** | | | | Extreme Police Violence  **N=101** | | | |
| --- | --- | --- | --- | --- | --- | --- | --- | --- | --- | --- | --- | --- |
| Covariate | OR | 95% CI | | p-value | OR | 95% CI | | p-value | OR | 95% CI | | p-value |
| **Age (N=2615)** | | | | | | | | | | | | |
| 18-24 | 0.76 | 0.56 | 1.02 | 0.07 | 2.60 | 1.77 | 3.82 | <0.001 | 8.45 | 2.46 | 29.01 | <0.01 |
| 25-34 | 0.80 | 0.61 | 1.04 | 0.09 | 3.13 | 2.21 | 4.44 | <0.001 | 11.23 | 3.42 | 36.88 | <0.001 |
| 35-44 | 0.78 | 0.59 | 1.04 | 0.09 | 2.47 | 1.70 | 3.60 | <0.001 | 10.74 | 3.21 | 35.93 | <0.001 |
| 45-54 | 0.78 | 0.85 | 1.03 | 0.08 | 1.74 | 1.17 | 2.56 | <0.01 | 4.53 | 1.26 | 16.34 | <0.05 |
| 55+ (ref) | - | - | - | - | - | - | - | - | - | - | - | - |
| **Gender (N=2598)** | | | | | | | | | | | | |
| Cisgender man | 0.90 | 0.75 | 1.09 | 0.29 | 1.01 | 0.82 | 1.25 | 0.92 | 2.76 | 1.79 | 4.25 | <0.001 |
| Cisgender woman (ref) | - | - | - | - | - | - | - | - | - | - | - | - |
| **Race (N=2615)** | | | | | | | | | | | | |
| Asian/Pacific Islander | 1.38 | 0.89 | 2.14 | 0.15 | 0.99 | 0.53 | 1.85 | 0.98 | 1.19 | 0.35 | 4.04 | 0.78 |
| Black/African American | 1.27 | 1.03 | 1.56 | <0.05 | 3.24 | 5.57 | 4.08 | <0.001 | 3.43 | 2.20 | 5.33 | <0.001 |
| Native American | 0.65 | 0.29 | 1.47 | 0.30 | 2.89 | 1.40 | 5.96 | <0.05 | 2.04 | 0.45 | 9.30 | 0.36 |
| Multiracial or other race | 1.04 | 0.67 | 1.60 | 0.87 | 2.55 | 1.62 | 4.01 | <0.001 | 2.44 | 1.04 | 5.79 | <0.05 |
| White (ref) | - | - | - | - | - | - | - | - | - | - | - | - |
| **Latino Ethnicity (N=2604)** |  |  |  |  |  |  |  |  |  |  |  |  |
| Latinx | 0.94 | 0.72 | 1.24 | 0.68 | 1.28 | 0.96 | 1.72 | 0.10 | 2.21 | 1.36 | 3.60 | <0.01 |
| Non-Latinx (ref) | - | - | - | - | - | - | - | - | - | - | - | - |
| **Sexual Orientation (N=2609)** | | | | | | | | | | | | |
| Bisexual | 0.68 | 0.43 | 1.08 | 0.10 | 1.90 | 1.25 | 2.90 | <0.01 | 1.62 | 0.74 | 3.57 | 0.23 |
| Gay/Lesbian/Homosexual | 0.90 | 0.55 | 1.49 | 0.69 | 1.42 | 0.84 | 2.40 | 0.19 | 0.55 | 0.13 | 2.36 | 0.42 |
| Sexual orientation not specified | 1.23 | 0.62 | 2.43 | 0.55 | 0.84 | 0.35 | 2.02 | 0.70 | 1.19 | 0.37 | 5.31 | 0.82 |
| Heterosexual/Straight (ref) | - | - | - | - | - | - | - | - | - | - | - | - |
| **Nativity (N=2604)** | | | | | | | | | | | | |
| Not born in USA | 1.29 | 0.96 | 1.72 | 0.09 | 0.70 | 0.47 | 1.02 | 0.06 | 0.46 | 0.18 | 1.16 | 0.10 |
| Born in USA (ref) | - | - | - | - | - | - | - | - | - | - | - | - |
| **Household Income (N=2606)** | | | | | | | | | | | | |
| < $19,999 | 1.83 | 1.33 | 2.52 | <0.001 | 2.69 | 1.83 | 3.95 | <0.001 | 3.25 | 1.66 | 6.38 | <0.001 |
| $20,000 – 39,999 | 1.21 | 0.91 | 1.62 | 0.19 | 2.36 | 1.67 | 3.32 | <0.001 | 1.28 | 0.62 | 2.64 | 0.51 |
| $40,000 – 59,999 | 1.18 | 0.89 | 1.56 | 0.26 | 2.01 | 1.42 | 2.83 | <0.001 | 1.65 | 0.84 | 3.23 | 0.14 |
| $60,000 – 79,999 | 1.24 | 0.92 | 1.66 | 0.15 | 2.01 | 1.41 | 2.87 | <0.001 | 1.37 | 0.66 | 2.83 | 0.40 |
| $80,000 – 99,999 | 0.88 | 0.63 | 1.23 | 0.46 | 1.51 | 1.01 | 2.25 | <0.05 | 1.63 | 0.78 | 3.44 | 0.20 |
| $100,000 + (ref) | - | - | - | - | - | - | - | - | - | - | - | - |
| **Education (N=2609)** | | | | | | | | | | | | |
| Did not complete high school | 2.45 | 1.20 | 4.61 | <0.05 | 3.09 | 1.47 | 6.49 | <0.01 | 8.05 | 2.48 | 26.14 | <0.001 |
| High school diploma or GED | 1.08 | 0.80 | 1.46 | 0.64 | 1.53 | 1.08 | 2.19 | <0.05 | 3.13 | 1.43 | 6.85 | <0.01 |
| Some college or technical school | 1.07 | 0.81 | 1.40 | 0.65 | 1.47 | 1.06 | 2.04 | <0.05 | 2.18 | 1.01 | 4.72 | <0.05 |
| College graduate | 1.06 | 0.81 | 1.38 | 0.69 | 1.32 | 0.96 | 1.82 | 0.09 | 1.89 | 0.88 | 4.06 | 0.10 |
| Graduate or professional degree (ref) | - | - | - | - | - | - | - | - | - | - | - | - |
| **Criminalized Behaviors** | | | | | | | | | | | | |
| Bought or sold drugs; used heroin or injected opiate drugs (ref: did not engage in behaviors) (N=2609) | 0.73 | 0.53 | 1.00 | <0.05 | 2.71 | 2.03 | 3.61 | <0.001 | 7.56 | 4.86 | 11.76 | <0.001 |
| Stole, robbed, or burglarized someone; taken money or goods (ref: did not engage in behaviors) (N=2614) | 0.73 | 0.47 | 1.13 | 0.16 | 3.05 | 2.09 | 4.46 | <0.001 | 9.28 | 5.59 | 15.41 | <0.001 |
| Assaulted someone; injured someone in a fight (ref: did not engage in behaviors) (N=2611) | 0.91 | 0.64 | 1.28 | 0.58 | 3.69 | 2.70 | 5.03 | <0.001 | 12.82 | 8.10 | 20.27 | <0.001 |

*Notes:* Transgender participants were excluded from the regression analysis due to the small sample size.
